# COVID-19 Infection Related Bowel Perforation

**DOI:** 10.7759/cureus.21830

**Published:** 2022-02-02

**Authors:** Dina Alnabwani, Nagapratap Ganta, Smriti Kochhar, Veera Jayasree Latha Bommu, Bassam Hasan, Michael Blake, Gustavo E Delaluz, Pramil Cheriyath

**Affiliations:** 1 Internal Medicine, Hackensack Meridian Ocean Medical Center, Brick, USA; 2 Medical Student, Hackensack Meridian Ocean Medical Center, Brick, USA; 3 Medical Student, Rowan School of Osteopathic medicine, Stratford, USA; 4 Infectious Diseases, Hackensack Meridian Ocean Medical Center, Brick, USA; 5 Hospital Medicine, Hackensack Meridian Ocean Medical Center, Brick, USA; 6 Pulmonary and Critical Care Medicine, Hackensack Meridian Ocean Medical Center, Brick, USA

**Keywords:** baricitinib, covid-19 infection, pneumoperitoneum, remdesivir, gastric perforation, bowel perforation, sars-cov-2

## Abstract

During an ongoing pandemic of severe acute respiratory syndrome coronavirus 2 (SARS-CoV-2), a novel virus, new discoveries about its complications and treatment are made every day. Bowel perforation is another rarely reported complication due to the virus itself leading to ischemia or can be due to the treatment with antiviral drugs that reduces the integrity of epithelial barriers. This makes the bowel more prone to perforation even in patients with no prior history of bowel disease. We report a case of bowel perforation in a 72-year-old male patient with severe COVID-19 infection.

## Introduction

The novel coronavirus disease, caused by the severe acute respiratory syndrome coronavirus 2 (SARS-CoV-2), has spread worldwide becoming a pandemic characterized mainly by respiratory symptoms. Gastrointestinal involvement, on the other hand, is rather common, with a prevalence of 10% to 50% [1]. With the start of the pandemic in December 2019, we have come a long way in inventing treatment options in preventing and curing the disease. To continue the invention of novel methods we need to understand the complications that arise due to the disease and its treatments. Gastrointestinal perforation in association with COVID-19 has been reported 25 times to date [2]. We report a case of bowel perforation in a 72-year-old male patient with severe COVID-19 infection with no past history of gastrointestinal disease.

## Case presentation

**Figure 1:**
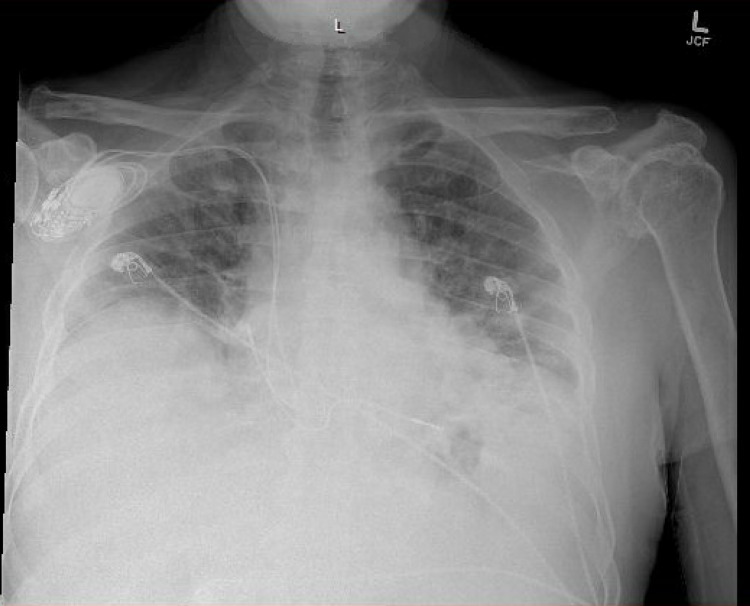
Chest X-ray on day one showing increased interstitial lung markings

A 72-year-old male patient presented to the emergency room (ER) with a cough, running nose, and malaise that started three to four days ago. The patient did not have any chest pain, palpitations, fever, diarrhea, loss of smell, or taste. The patient has received one dose of Moderna vaccine six months prior to admission but did not complete his vaccination series. He was placed on supplemental oxygen with the goal to keep oxygen saturation above 90%. Other vitals were stable within normal limits. ​​Past medical history included benign prostatic hypertension (BPH), diabetes mellitus type 2, ischemic cardiomyopathy, hepatitis C, ascites. Past surgical history includes a cardiac pacemaker insertion. Initially, COVID-19 was treated with intravenous Remdesivir and intravenous dexamethasone 6 mg. Later Baricitinib was added for 14 days. For deep vein thrombosis (DVT) prophylaxis, he was administered enoxaparin and a sequential compression device. Other medications that he was on at home were sitagliptin, tamsulosin and finasteride, and metformin but on the first day of hospitalization, aspirin and atorvastatin were added. With day one lab abnormalities (Table 1) showing, total bilirubin 2.2 mg/dL (0.3-1.0 mg/dL) and aspartate aminotransferase 78 U/L (0-40 U/L), while other labs were normal. The chest x-ray from day one showed increased interstitial lung markings that may be related to viral pneumonia or edema (Figure 1).

**Figure 2:**
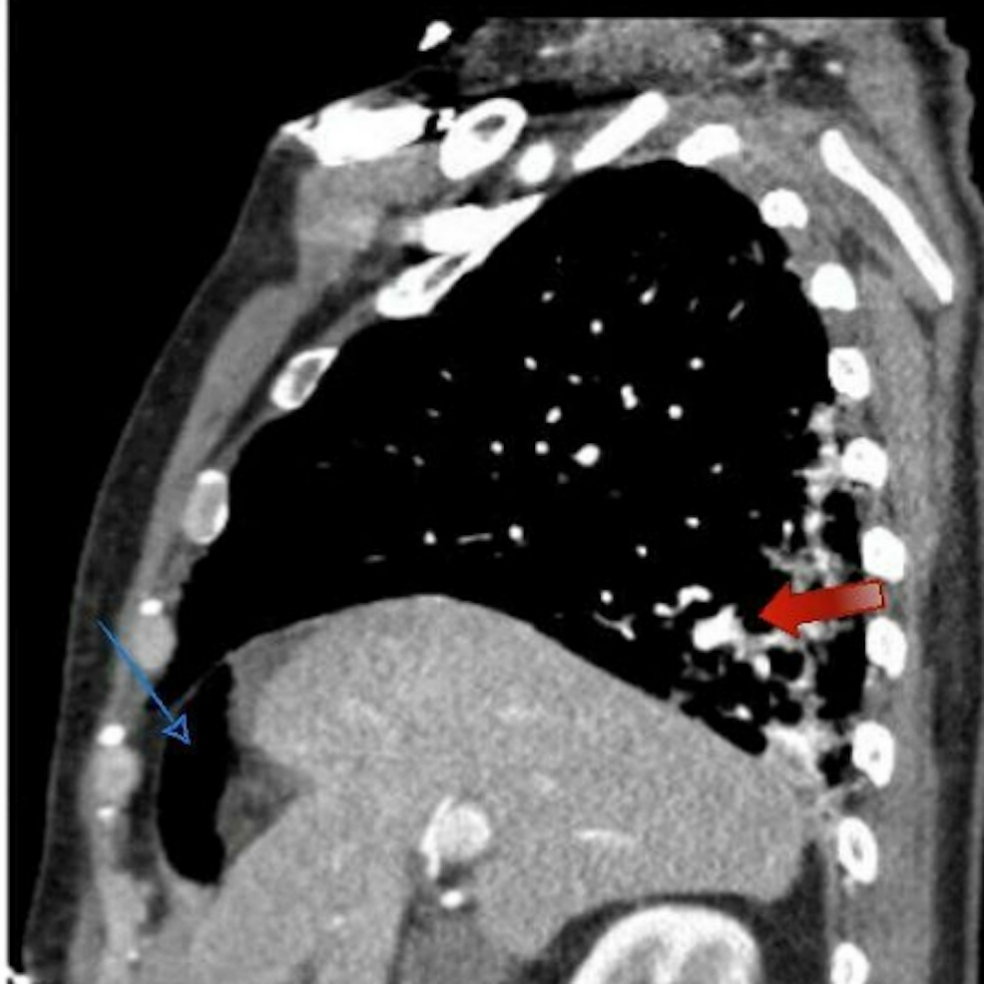
Chest CT without contrast sagittal view showing moderate patchy bilateral airspace disease most pronounced at the lung bases with areas of bronchiectasis (red arrow), cystic changes, and pneumoperitoneum (blue arrow)

Over the course of the patient's hospital stay the goal was to improve the impaired respiration but as soon as he was weaned off oxygen therapy, he would desaturate into 70%. This continued for 14 days of hospital stay until the patient's condition worsened with 10/10 epigastric abdominal pain with mild ecchymosis in the epigastric region and nausea but no accompanying diarrhea or vomiting. The patient’s oxygen saturation (SpO2) was 80%, on 50L of nasal high flow oxygen (Optiflow). The vitals from that day includes a temperature of 98.1 ℉, heart rate (HR) 61 beats per minute, respiratory rate (RR) of 22 breaths per minute, blood pressure (BP) of 118/57 mm Hg. The repeat labs (Table 1) showed creatinine 0.95 mg/dL (0.7 to 1.3 mg/dL), lactic acid 3.7 mg/dL (4.5 to 19.8 mg/dL), lactate dehydrogenase (LDH) 383U/L (91 - 200 U/L)​​. To rule out pulmonary embolism, computer tomography (CT) of the chest without contrast was ordered which showed moderate patchy bilateral airspace disease most pronounced at the lung bases with areas of bronchiectasis and cystic changes compared to prior and pneumoperitoneum and free fluid were also noted (Figure 2). 

**Table 1:** Comparing patient’s labs over the course of his hospital admission WBC: white blood cell, BUN: blood urea nitrogen

Labs	Day 1	Day 14	Day of death
WBC (cells/mL3)	3,300	3,500	6,500
Platelets (cells/mcl)	67,000	137,000	47,000
BUN (mg/dl)	26	46	99
Sodium (Na) (mEq/L)	129	135	134
Potassium (K) (mEq/L)	5.4		5.3

**Figure 3:**
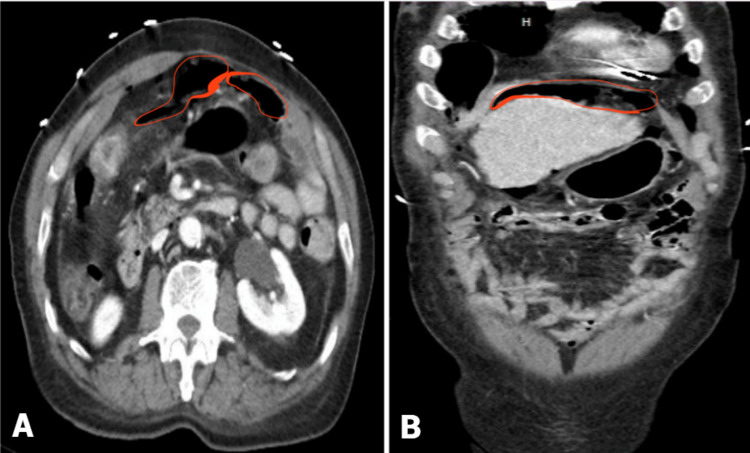
CT of the abdomen and pelvis with intravenous contrast A: transverse view, B: coronal view showing moderate volume pneumoperitoneum with trace free fluid in the upper abdomen (red marked areas) compatible with perforation

Results from this study led to ordering computerized tomography (CT) of the abdomen and pelvis with contrast which showed moderate volume pneumoperitoneum with trace free fluid in the upper abdomen compatible with perforation, suspected from the distal stomach (Figure 3).

The patient was taken for an immediate diagnostic laparoscopy with the repair of bowel perforation with a ​​graham patch, abdominal washout, and drain placement and was taken again to the operating room (OR) two days later for Graham patch repair. Over the next 15 days the patient remained intubated, with vital signs as follows and labs showing red blood cells (RBC) 2.74 million/mm3 (5.1 to 6.1 million/mm3), hemoglobin 7.8 g/dl (14 to 18 g/dl), hematocrit 26.7% (36.0 - 53.0 %) Creatinine 2.17 mg/dl (0.61 - 1.24 mg/dL), eGFR 30 (> 60 mL/min/1.73m*2), Albumin 1.8 g/dl (3.5 - 5.0 g/dL), AST 191 U/L (10 - 42 U/L), ALT 108 U/L (10 - 60 U/L), and other labs are mentioned in Table 1.

**Figure 4:**
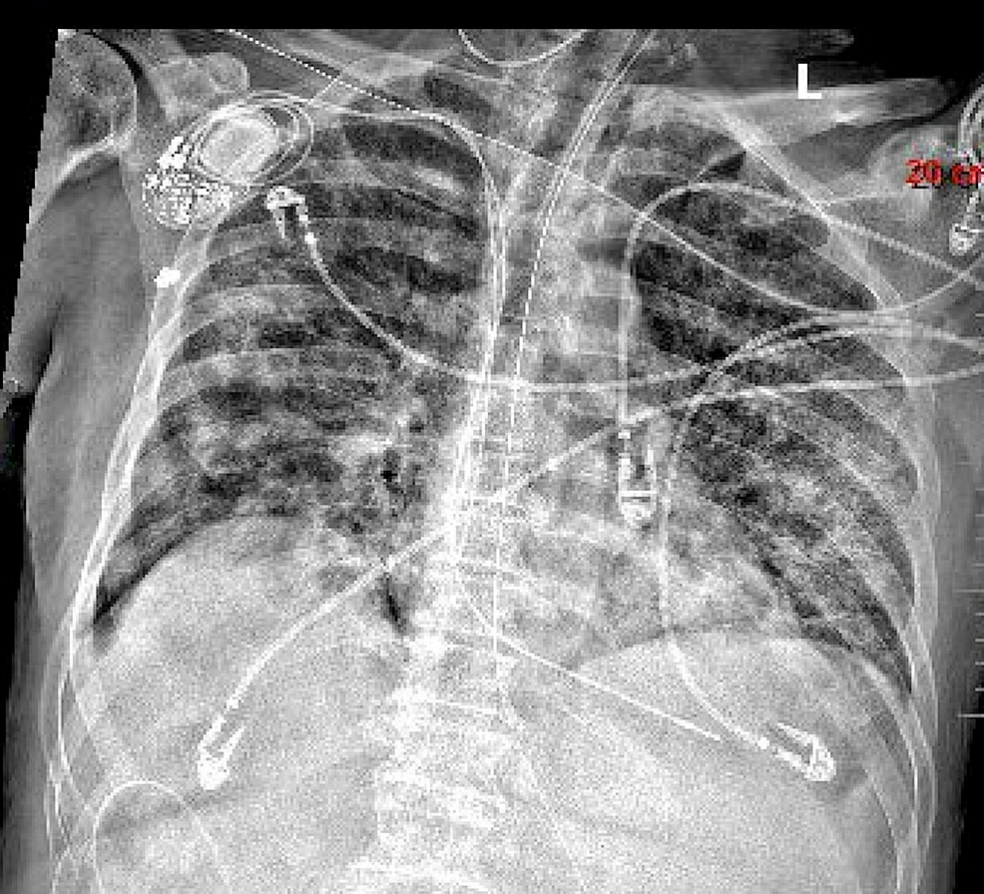
Chest x-ray on the day of patient’s death showing elevation of the right hemidiaphragm and worsening patchy bilateral airspace disease

The patient’s x-ray from the same day showed low volumes with persistent elevation of the right hemidiaphragm and worsening patchy bilateral airspace disease (Figure 4). Due to lack of improvement in the patient’s condition despite aggressive medical interventions, Based on further discussion with the family about goals of care, the patient was transitioned to hospice care and passed away the same day.

## Discussion

SARS-COV-2, more commonly known as COVID-19 is known to cause upper-respiratory symptoms such as fever, cough, dyspnea, and respiratory illness, representing the most common manifestations. In addition, headache, dizziness, generalized weakness, vomiting, and diarrhea were observed [1]. In a study done by Kaafarani et al., on 141 intensive care unit (ICU) patients, 45% of the patients had gastrointestinal symptoms such as nausea, vomiting, abdominal pain, diarrhea [3]. The study also noted several other complications including necrosis of the bowel, ileus, Ogilvie-like syndrome, etc were reported in high incidence with critically ill patients [3].

As seen in the above patient who had gastric perforation as a complication secondary to COVID-19 after being admitted to the ICU. The concerning signs of gastric perforation are sudden, severe chest or abdominal pain [4]. The effect of SARS-COV-2 virus on the GI system can be explained by different mechanisms. Angiotensin-converting enzyme 2 (ACE2) is a receptor for SARS-CoV-2 virus which is commonly found in lung, intestinal and vascular tissue. The ACE2 receptor becomes the primary site for entry by SARS-CoV-2 making the cells targets of viral entry and replication. The continuous insult on the tissue wall leads to a vicious circle of destruction and repair, eventually culminating in progressive damage [4]. Similarly, the presence of SARS-CoV-2 within the intestinal tissue may cause an inflammatory response, thereby weakening the intestinal wall leading to bowel perforation. 

The virus may also induce ischemia secondary to massive endothelial dysfunction, widespread coagulopathy, and complement-induced thrombosis can lead to the development of systemic microangiopathy and thromboembolism [5]. While these factors can lead to the weakening of the gastrointestinal wall, it is also important to understand the role immunosuppressive or anti-inflammatory agents may have in an impairing inflammatory response [2], as they reduce systemic inflammation before it overwhelmingly results in multi-organ dysfunction [6]. 

The treatment for COVID-19 can not only mask the symptoms of sepsis but can also be another cause of gastric perforation. A study shows the increased risk of JAK-2 inhibitors such as Baricitinib can lead to increased GI perforation with other exaggerating factors including older age, other gastrointestinal (GI) conditions, and the use of prednisone ⩾ 7.5 mg/day [7]. Our patient was being treated with 12 mg/day of intravenous Dexamethasone and Baricitinib daily for 13 days before he started complaining of excruciating pain with an increase in white blood cell count, lactic acid, and hypotension from the gastric perforation. The combined effect of both COVID-19 infection and treatment lead to gastric perforation in this patient.

Similar to the case by Cyr et al., our case also highlights a significant clinical issue regarding Remdesivir. In vitro, it inhibits multi-drug resistance associated with protein 4 (MRP4), which partakes in cell proliferation. Our patient finished a ten-day course of Remdesivir three days prior to gastric perforation. This may lead to reduced integrity of the epithelial barrier, causing bowel perforation [8].

Radiological findings especially on CT scan is the mainstay of diagnosing GI perforation. The most specific findings are segmental bowel wall thickening, focal bowel wall defect, or bubbles of extraluminal gas concentrated in close proximity to the bowel wall [9]. Treatment of GI perforation is mainly surgical in order to improve survival [10]. However, in selected cases where there are no active signs of peritonitis, abdominal sepsis, or having sealed perforation, conservative treatment is an acceptable management strategy [11]. 

## Conclusions

GI manifestations are common in patients with COVID-19, however, it has rarely been reported in the literature. To the best of our knowledge, this is the first case with bowel perforation in a patient being treated for COVID -19 with Baricitinib. Severe and critically ill COVID-19 patients seem to be at a higher risk of this complication. It has a variable presentation in patients with COVID-19 ranging from incidental findings discovered only radiographically to acute abdomen. A high index of suspicion is required in order to manage those patients further and thus, improve their outcomes.
